# *In Vitro* Investigation of Self-Assembled Nanoparticles Based on Hyaluronic Acid-Deoxycholic Acid Conjugates for Controlled Release Doxorubicin: Effect of Degree of Substitution of Deoxycholic Acid

**DOI:** 10.3390/ijms16047195

**Published:** 2015-03-31

**Authors:** Wen-Hao Wei, Xue-Meng Dong, Chen-Guang Liu

**Affiliations:** College of Marine Life Science, Ocean University of China, Yusan Road No. 5, Qingdao 266003, China; E-Mails: weiwen_1980@163.com (W.-H.W.); dxm090981@163.com (X.-M.D.)

**Keywords:** hyaluronic acid, deoxycholicacid, nanoparticles, doxorubicin

## Abstract

Self-assembled nanoparticles based on a hyaluronic acid-deoxycholic acid (HD) chemical conjugate with different degree of substitution (DS) of deoxycholic acid (DOCA) were prepared. The degree of substitution (DS) was determined by titration method. The nanoparticles were loaded with doxorubicin (DOX) as the model drug. The human cervical cancer (HeLa) cell line was utilized for *in vitro* studies and cell cytotoxicity of DOX incorporated in the HD nanoparticles was accessed by the 3-(4,5-dimethylthiazol-2-yl)-2,5-diphenyltetrazolium bromide (MTT) assay. In addition, cellular uptake of fluorescently labeled nanoparticles was also investigated. An increase in the degree of deoxycholic acid substitution reduced the size of the nanoparticles and also enhanced their drug encapsulation efficiency (EE), which increased with the increase of DS. A higher degree of deoxycholic acid substitution also lead to a lower release rate and an initial burst release of doxorubicin from the nanoparticles. In summary, the degree of substitution allows the modulation of the particle size, drug encapsulation efficiency, drug release rate, and cell uptake efficiency of the nanoparticles. The herein developed hyaluronic acid-deoxycholic acid conjugates are a good candidate for drug delivery and could potentiate therapeutic formulations for doxorubicin–mediated cancer therapy.

## 1. Introduction

Polymeric nanoparticles prepared from natural or synthetic polymers have become an important area of research in the field of drug delivery due to their controlled release properties and the protection they offer to the compound of interest [1]. In recent decades, significant efforts have been devoted to develop nanoparticles based on natural polymers such as albumin, gelatin, alginate, collagen or chitosan [2,3]. Among these polymers, polysaccharides are the most popular polymeric materials for the preparation of nanoparticles for drug delivery due to their stability, biodegradability and non-toxicity [4,5]. These nanoparticles can be prepared by covalent crosslinking, ionic crosslinking, polyelectrolyte complexation or self-assembly of hydrophobically modified polysaccharides (HMP). Numerous studies have been carried out to investigate nanoparticles based on HMP because of their self-aggregation characteristics [6,7]. HMP in aqueous media is driven by intra-and/or intermolecular hydrophobic interactions to form nanoparticles with hydrophobic core and hydrophilic shell. Hydrophobic drugs can thus be easily entrapped within the inner core by hydrophobic interactions [8,9].

Hyaluronic acid (HA), a linear polysaccharide formed by alternating d-glucuronic acid (GlcUA) and *N*-acetyl-d-glucosamine (GlcNAc) units, are biopolymers belonging to glycosaminoglycans [10]. HA is widely found in living cells and is abundant in the extracellular matrix of tissues including skin, vessels, cartilage and brain [11,12]. The immune neutrality of HA make it an excellent material for tissue engineering and drug delivery systems [13,14,15,16,17]. Furthermore, HA plays important roles in regulating cell adhesion and motility, and mediating cell proliferation and differentiation through its receptors, mainly the CD44 and RHAMM receptors. CD44 and RHAMM were over expressed in many types of tumors, for example ovarian tumor cells, colon cancer, lung cancer, stomach cancer, acute leukemia, and other cancers [18,19]. As a result, malignant cells with high metastatic potential often show enhanced binding and internalization of HA. The high tumor specificity of HA prompted the design of target-specific drug delivery vehicles for various therapeutic agents [20,21]. Several bioconjugates of HA and cytotoxicity agents were shown to have an increased drug uptake into tumor cells [22,23]. Liposomes decorated with HA oligomers can preferentially bind to and be internalized by certain types of cancer cells whose surfaces include relatively large numbers of CD44 receptors [24]. Recently, HA has been extensively used as a targeting moiety in HA self-assembled nanoparticles for cancer targeting delivery of chemotherapy drugs [25,26,27,28].

In a previous study we developed hyaluronic acid-deoxycholic acid (HD) conjugates, which spontaneously form self-aggregates in an aqueous environment with a low critical aggregation concentrations and small particle sizes [29]. Molecular weight and degree of substitution of hydrophobic groups are important parameters that influence physicochemical, biological and pharmaceutical properties of nanoparticles. For example, the degree of betaine substitution of chitosan *N*-betainates was crucial for cellular uptake, cytotoxicity and transfection efficiency of chitosan *N*-betainates/DNA nanoparticles against COS-7 and MDA-MB-468 cells [30]. In the present work, we extended our earlier approach on HD conjugates for nanoparticle formulation of anticancer drugs and investigated the effects of the degree of substitution (DS) of deoxycholic acid (DOCA) on the *in vitro* pharmaceutical characteristic of drug-loaded HD nanoparticles.

## 2. Results and Discussion

### 2.1. Preparation and Characterization of Hyaluronic Acid-Deoxycholic Acid (HD) Self-Assembled Nanoparticles

**Figure 1:**
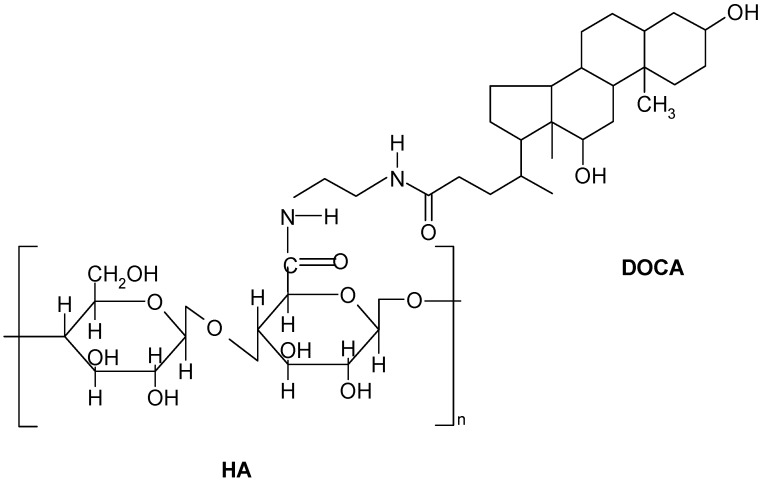
Structure of HA-DOCA (HD) conjugate. HD conjugate was synthesized by coupling the carboxylic groups of HA with *N*-(2-aminoethyl) deoxycholylamide (DOCA-NH_2_) in the presence of EDC.

The structure of the amphiphilic HD conjugate is shown in Figure 1. The amphiphilic HA derivatives were prepared by chemically coupling hydrophobic DOCA-NH_2_ onto hydrophilic HA through the formation of an amide bond with 1-ethyl-3-(3-dimethylaminopropyl)carbodiimide (EDC). The presence of DOCA in HD was verified by the characteristic peaks of DOCA at 0.67, 0.85 and 0.99 ppm appearing in the ^1^H NMR spectra [29]. Various HD conjugates with different amounts of DOCA were prepared by changing the feed ratio of HA to DOCA. The degree of substitution of DOCA ranges from 5.9 to 9.4 per HA molecule as measured by a titration method. The molecular weight of this HD conjugate linearly increased with the increasing of DS. When the amphiphilic conjugates were dispersed in distilled water and sonicated, the conjugates formed self-assembled nanoparticles with diameters of 208–1044 nm, as determined by dynamic light scattering. The molecular weight, average mean diameters and DS are summarized in Table 1. Notably, the size of nanoparticles decreases with increasing of DS, indicating that a densely packed hydrophobic core was formed due to the enhanced hydrophobic interaction.

**Table 1:** General properties of HD conjugate in distilled water.

Sample ^a^	Feed Ratio ^b^	Mn ^c^	DS	X ^d^ (%)	*d* (nm)
HD6	1:10	18,925	5.9	12.3 ± 1.2	1044 ± 70.2
HD7	1:20	19,594	7.6	15.3 ± 2.3	447 ± 31.8
HD9	1:40	20,304	9.4	18.2 ± 3.1	208 ± 20.1

^a^ HA-DOCA (HD) conjugates, where the number indicates the DS of DOCA; ^b^ Mole ratio of A:EDC:DOCA-NH_2_; ^c^ Number-average molecular weight, estimated from the titration results; and ^d^ Weight fraction of DOCA in a HA molecule.

### 2.2. Preparation and Characterization Doxorubicin (DOX)-Loaded HD Nanoparticles

HD nanoparticles loaded with DOX were prepared by dialysis. To obtain a high amount of DOX incorporation, triethylamine was added to the organic solvent to remove hydrochloride from DOX·HCl [31]. DOX was incorporated within self-assembled HD nanoparticles by hydrophobic interactions between DOX and the hydrophobic groups in the HD conjugate nanoparticles. Particle size of DOX-loaded HD9 nanoparticles was shown in Table 2. Both the particle size and PDI gradually increased depending on the drug content. Drug loading efficiency is summarized in Table 3. The loading content increased with an increase of the feed-to-weight ratio of initially added amount of DOX to the HD conjugate. However, the variation of loading efficiency was inversely proportional to that ratio. Table 3 shows that when the weight percentage of initial added DOX to HD6 nanoparticles increased (from 5% to 20%), the loading efficiency of HD6 nanoparticles decreased (from 95.00% ± 2.51% to 57.50% ± 3.82%). At ratios higher than 20%, the loading efficiency significantly decreases to about 55% due to severe precipitation of the DOX-loaded HD nanoparticles solution after sonication. This behavior can be explained by the enhanced hydrophobicity at higher DOX loadings, which then destroys the balance between hydrophobic and hydrophilic regions within amphiphilic molecules. Self-assembly of the DOX-loaded HD conjugates would thus be hampered due to the high hydrophobicity of DOX.

**Table 2:** Particle size of doxorubicin (DOX)-loaded HD9 nanoparticles (*n* = 3).

Loading Amount of DOX (wt. %) ^a^	0	5	10	15
Particle size (nm)	208 ± 20.1	212 ± 23.1	220 ± 27.1	228 ± 30.1
PDI ^b^	0.290	0.300	0.350	0.367

^a^ the weight percentage of initial added DOX to HD nanoparticles; and ^b^ polydispersity index.

**Table 3:** Characterizations of DOX-loaded HD nanoparticles (*n* = 3).

Samples	Loading Amount of DOX (wt. %) ^a^	Loading Content of DOX (%)	Loading Efficiency (%)
HD6	5	4.75 ± 0.21	95.00 ± 2.51
HD6	10	7.88 ± 0.87	78.75 ± 6.13
HD6	20	11.58 ± 1.12	57.50 ± 3.81
HD7	5	4.89 ± 0.53	98.08 ± 9.42
HD9	5	4.97 ± 0.57	99.33 ± 10.91

^a^ weight percentage of initial added DOX to HD nanoparticles.

From Table 3, it is also obvious that the loading efficiency and loading content are significantly affected by the degree of substitution of DOCA in HD. When the same initial amount of DOX (5 wt. %) was added, the loading efficiencies of DOX in the HD6, HD7 and HD9 nanoparticles were 95%, 98% and 99.3%, respectively. The DOX loading content of the three HD nanoparticles increased from 4.75% to 4.97%, reflecting the increase in the degree of substitution of DOCA. With increasing amounts of DOCA groups in the HD conjugates, the concentration of hydrophobic domains in the HD nanoparticles and the mean aggregation number of DOCA groups per single hydrophobic domain increase progressively. Thereby, more sites for hydrophobic interaction with DOX are accessible in the hydrophobic inter-cores of HD nanoparticles with higher DS, which can encapsulate a higher amount of DOX.

### 2.3. In Vitro Release of Doxorubicin

**Figure 2:**
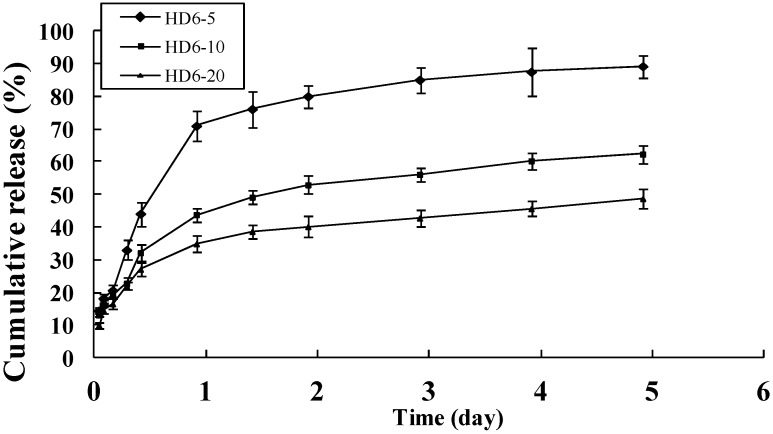
Release profile of DOX from HD6 nanoparticles with 5%, 10% and 20% weight percentage of initial added drug to HD conjugates, mean ±Standard Deviation (S.D.), *n* = 3.

**Figure 3:**
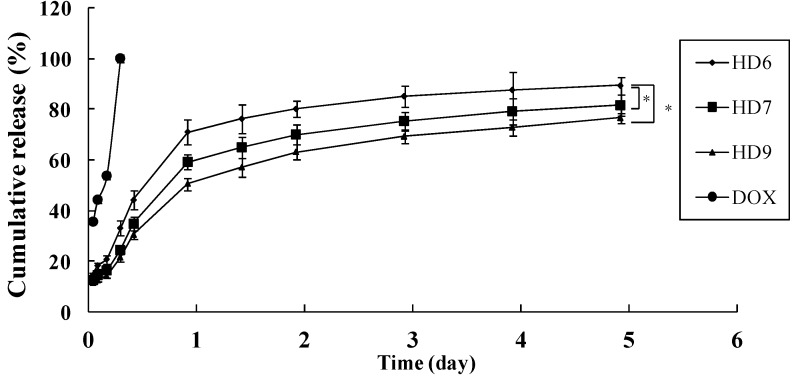
Release profile of unloaded DOX from the dialysis bag and DOX from HD nanoparticles with different DS. The DS of HD6, HD7 and HD9 was 5.9, 7.6 and 9.4, respectively, mean ± S.D., *n* = 3, * *p* < 0.05.

DOX is one of the most effective anticancer agents. In clinical practice, however, high incidence of adverse reactions, including neurotoxicity, myelosuppression, and allergic reactions have been reported [32,33,34,35]. To evaluate the potential of HD nanoparticles as carrier of DOX, the releasing pattern of DOX from HD nanoparticles was assessed at 37 °C in physiological PBS buffer. As shown in Figure 2 and Figure 3, the releasing behavior of DOX exhibits a triphasic pattern characterized by a fast initial release, followed by a slower and continuous release. Figure 2 shows the release profile of DOX from DOX-loaded HD nanoparticles (HD6-5, HD6-10, HD6-20) with different initial added amount of drug, where the DOX content is in the range of 4.75% to 11.58% (wt. %). It can be seen that the drug release from the HD6-5, HD6-10, and HD6-20 nanoparticles displayed an initial burst of 20.7%, 19.2% and 16.6% loss within 5 h, respectively, which is followed by an apparent first-order release. After 5 days, the cumulative drug release approached 50%–90% of the initial loading. Notably, the release rate of DOX from the nanoparticles depends on the initial amount of added drug (Figure 2). A possible reason is that at small DOX loadings the nanoparticles are less densely packed which favors the diffusion inside/outside and leads to an increased drug release. Alternatively, the hydrophobic drug encapsulated in the nanospheres could partially crystallize at higher drug concentrations [36]. At lower drug loadings, the hydrophobic drug forms a molecular dispersion within the nanoparticles. The crystallized drug is expected to dissolve and diffuse slower into the outer aqueous phase relative to such a molecular dispersion.

Figure 3 shows the cumulative amount of DOX released from HD nanoparticles with different DS (*i.e*., the amount of DOCA groups in HD conjugates) of 5.9, 7.6 and 9.4, respectively. The initial weight ratio of DOX to HD conjugates was 5%. Depending on the DS, HD nanoparticles showed different releasing profiles. The release rate of DOX from the nanoparticles decreased with increasing DS. The HD nanoparticles with a DS of 9.4 displayed the slowest drug release of 70% within 5 days, while nanoparticles with a DS of 5.9 presented a faster release of 90% within 5 days. The reason is that more hydrophobic microdomains of nanoparticles are formed due to higher DS of DOCA in the HD conjugates. As a result, less DOX was released from the nanoparticles because of enhanced interaction between the hydrophobic drug and the hydrophobic core of the nanoparticles. This experiment can be used to adjust the drug release rate by changing the DS of DOCA in HD nanoparticles in order to optimize the formulation of various drugs to meet the therapeutic needs.

**Figure 4:**
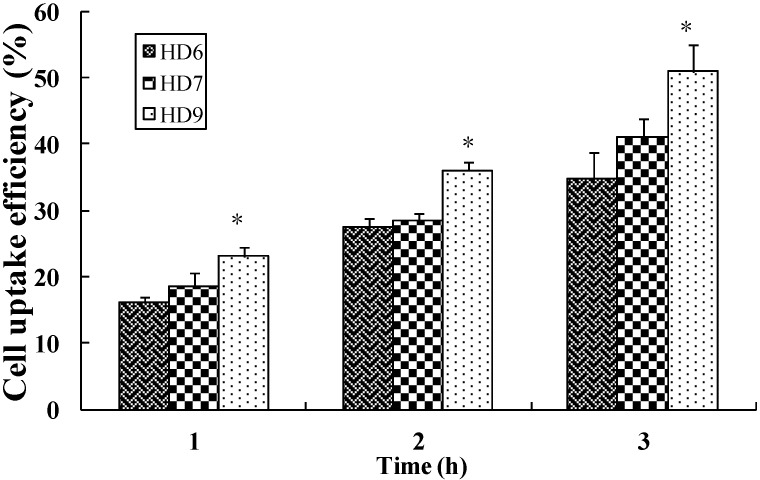
The cellular uptake efficiency of fluorescent HD nanoparticles with different DS in HeLa cells. The nanoparticles concentration is 0.1 mg/mL, mean ± S.D., *n* = 3, * *p* < 0.05.

### 2.4. Cellular Uptake of HD Nanoparticles

Characterization of the cellular uptake of fluorescent HD nanoparticles showed a significant effect of the DS of the HD nanoparticles. The cellular uptake efficiency of FITC-labeled HD nanoparticles with three different DS is expressed as the percentage of the number of the internalized nanoparticles *versus* the total number of nanoparticles applied. Figure 4 shows that the uptake of nanoparticles by cells increased with an enhanced degree of substitution of DOCA to HA. The cellular uptake efficiency was increased from 16.12% for the HD6 to 23.01% for the HD9, 27.37% for the HD6 to 35.99% for the HD9, and 34.66% for the HD6 to 50.91% for the HD9, at the incubation time of 1, 2 and 4 h respectively. It was shown that the higher DOCA content in the HD led to higher cell uptake efficiency. The possible reason is that at a higher DS of DOCA leads to a smaller average diameter of the HD, which is then easier internalized by the cells via CD44 receptor-mediated endocytosis. On the contrary, a lower DS leads to larger particle sizes, which hamper effective binding to CD44 receptors due to steric hindrances.

**Figure 5:**
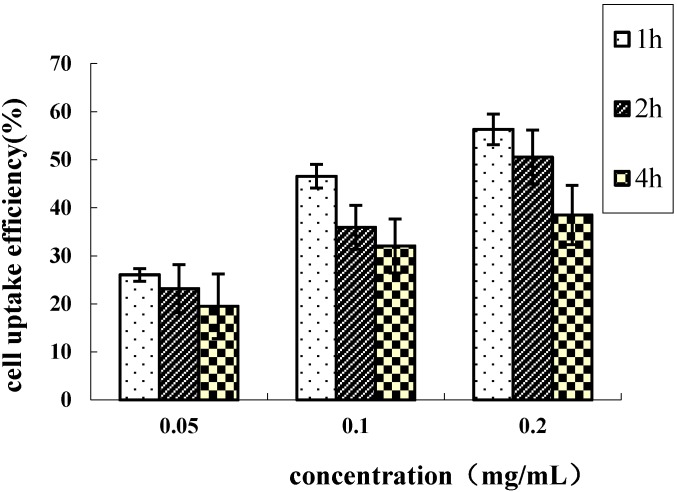
The effects of particle suspension concentration on the HeLa uptake efficiency of the HD9 nanoparticles, mean ± S.D., *n* = 3.

On the other hand, nanoparticle uptake was both concentration- and time- dependent. The uptake efficiency was higher at lower nanoparticle doses. A concentration of 0.2 mg/mL resulted in a 1.31-, 1.45- and 1.49-fold higher uptake-rate than a concentration of 0.05 mg/mL for the incubation times of 1, 2 and 4 h, respectively (Figure 5). At a lower concentration range the nanoparticles could be transported through nonspecific receptor mediated endocytosis. At higher nanoparticle concentrations, however, the cells probably reach a saturation uptake due to CD44 receptor-mediated endocytosis, and hence the uptake efficiency might be reduced. Figure 5 also shows that longer incubation times increase the efficiency of uptake. At a nanoparticle concentration of 0.2 mg/mL the cellular uptake efficiency after 4 h was 1.98-fold higher when compared to the first hour. The cellular uptake increased linearly with the incubation time and particle concentration. The slower increase of uptake beyond 1 h of incubation could be due to the limited saturation level.

### 2.5. In Vitro Cytotoxicity Assay

Blank nanoparticles did not cause significant cytotoxicity against HeLa cells up to a total nanoparticle dose of 200 µg/mL within 72 h. The concentration of blank nanoparticles applied in this experiment was higher than the maximum concentration of drug-loaded nanoparticles estimated in the cytotoxicity experiment as calculated from the drug loading content. As expected, the cytotoxicity of blank HD nanoparticles did not increase with the increase in incubation time, which correlated to the composition of the nanoparticles.

**Figure 6:**
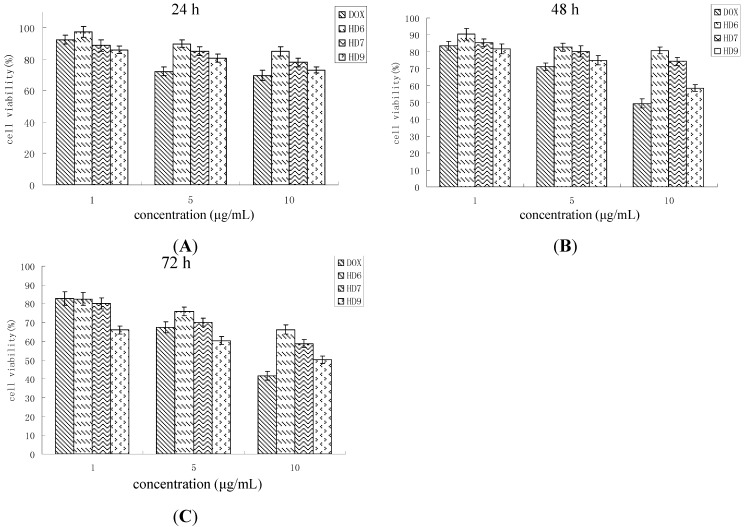
Cancer cell cytotoxicity of free DOX and DOX-HD nanoparticles on HeLa cells within the incubation times of 24 h (**A**); 48 h (**B**); 72 h (**C**), mean ± S.D., *n* = 3.

The primary ligands of the CD44 receptor is HA and targeting of anti-cancer agents to tumor cells can be accomplished by receptor-mediated uptake of anticancer agents conjugated to HA. For example, *N*-(2-hydroxypropyl) methacrylamide copolymer-DOX conjugates containing HA significantly increased the efficiency of endocytosis by cancer cells [20]. In this study, the uptake of drug-loaded HD nanoparticles into HeLa cells may occur by a similar pathway (Figure 6); the cytotoxicity of DOX-HD nanoparticles was higher against HeLa cells when compared to free DOX. To this end, the possible mechanism of nanoparticles uptake are still not known with certainty and require further investigations.

At high concentration (10 µg/mL), drug-loaded HD nanoparticles show lower cytotoxicity when compared to free DOX. For example, cytotoxicity of free DOX increased with increasing concentration in a cell viability of 41.67% after three days. The cell viability of HD nanoparticles containing the same amount of DOX was 50.24% after the same incubation time. DOX encapsulation into the cores of the nanoparticles may therefore limit the DOX release into the cytoplasm which reduces the direct interaction of DOX and HeLa cells. According to the release profile, the accumulative release amount of DOX from HD6 nanoparticles loaded with 10% of the drug only reaches 56.13% within 72 h, so the DOX concentration may be too low to trigger a cytotoxicity activity comparable to free DOX. This indicates that the drug-loaded HD nanoparticles could be more cytotoxic than free DOX at a longer incubated period.

When nanoparticle suspensions with same amount of DOX were added to cell culture medium, HD9 exhibited slightly more cytotoxic effects than HD6 and HD7. The difference in the efficiency of uptake observed for the different types of nanoparticles explained their difference in cytotoxicity. Nanoparticles with a higher DS and thus, a smaller size could be taken up more efficiently by the HeLa cells than nanoparticles with a larger particle size (Figure 4). Indeed, the HD9 nanoparticles, which exhibited a higher uptake efficiency by the cells than the other two types of nanoparticles, also exerted the highest cytotoxicity among all investigated nanoparticles.

## 3. Materials and Methods

### 3.1. Materials

Hyaluronic acid (HA) (average *M*r = 16,600 Da) was purchased from Freda Biochemical company (Shandong, China). Deoxycholic acid (DOCA), anhydrous dimethylformamide (DMF), 1-ethyl-3-(3-dimethylaminopropyl) carbodiimide (EDC), terahydrofuran (THF), *N*-hydroxysuccinimide (NHS), *N*,*N*-dicyclohexylcarbodiimide (DCC), anhydrous formamide,triethylamine (TEA), trypsin-EDTA solution, 3-(4,5-dimethylthiazol-2-yl)-2,5-diphenyltetrazolium bromide (MTT) and fluorescein isothiocyanate (FITC) were obtained from Sigma (St. Louis, MO, USA) and used without further purification. Doxorubicin hydrochloride (DOX·HCl) was purchased from Xingchang Pharmacy Co., Ltd. (Zhejiang, China). Fetal bovine serum, RPMI-1640 medium were obtained from Gibco BRL (Gaithersberg, MD, USA). All other chemicals were analytical grade and used as received.

### 3.2. Synthesis of N-Deoxycholyl-ethylenediamine (DOCA-NH_2_)

Firstly, the carboxylic group of deoxycholic acid was activated, as reported previously [37]. Briefly, DOCA (3.54 g) was mixed with DCC (2.40 g) and NHS (1.48 g) in 30 mL of DMF. The feed molar ratio of DOCA, DCC and NHS were 1:1.2:1.2. The concentration of DCC and NHS were slightly higher than DOCA in order to activate DOCA completely. The mixture was allowed to react for 12 h at room temperature under a nitrogen atmosphere and the precipitated dicyclohexylurea was removed by filtration. The filtered solution was poured into 300 mL of hexane and the remaining NHS was dissolved in the hexane. The succinimido DOCA precipitate was filtered off and washed thoroughly with hexane, followed by vacuum-drying (DZF, Chemat, Northridge, CA, USA) at room temperature. The succinimido DOCA was stored at −20 °C before use.

The *N*-deoxycholyl-ethylenediamine (DOCA-NH_2_) was synthesized by introducing ethylenediamine to the succinimido DOCA [31]. The succinimido DOCA (5 g, 12.7 mmol) was dissolved in DMF (20 mL) in a 250 mL beaker and the solution was slowly added dropwise into ethylenediamine solution (70 mL, 1mol) with a separatory funnel (34731-00, Cole-Parmer, Vernon Hills, IL, USA). The free mole ratio of succinimido DOCA and ethylenediamine was about 1:100. After reacting for 6 h, the mixture was precipitated in excess of distilled water. The filtered precipitation was carefully washed three times with distilled water and vacuum-dried at room temperature to obtain a white DOCA-NH_2_ powder.

### 3.3. Preparation of HA-DOCA (HD) Conjugate

HA (0.1 g) was dissolved in formamide (5 mL) with gentle magnetic stirring, in a 20 mL beaker, at room temperature. Different amounts (the mole ratio to HA was 5:1, 10:1, 15:1, respectively) of EDC were thereafter added to the HA solution. Different amounts of DOCA-NH_2_ (the mole ratio to HA was 10:1, 20:1, 40:1, respectively) were dissolved in DMF (5 mL) in a 20 mL beaker, by gentle heating (at 50 °C) and added into the mixed solution of HA and EDC. The resulting solution was thereafter cooled at room temperature and then stirred under a nitrogen atmosphere for 24 h. The EDC acted as an activator, allowing formation of the amide linkage between the carboxyl group of the HA and the amine group of the deoxycholyl-ethylamine. After the mixtures precipitated in an excess of cold acetone (analytical reagent, Sigma), the product were carefully washed three times with acetone to remove excess DOCA-NH_2_, followed by drying in a vacuum. The chemical structures of the synthesized HA-DOCA conjugates were analyzed by ^1^H NMR in CDCl_3_ using a 500-MHz NMR (UNIYTINOVA-500 NMR, VARIAN, Palo Alto, CA, USA) at 25 °C. Various HA-DOCA conjugates were prepared by controlling the free mole ratios of DOCA-NH_2_ to HA (*i.e.*, HD6, HD7, and HD9. The number designates the degree of substitution of DOCA). The degree of substitution (DS), defined as the number of DOCA per one HA molecules, was determined by a titration method.

### 3.4. Preparation and Characterization Doxorubicin-Loaded HD Nanoparticles

The doxorubicin-loaded HD nanoparticles were fabricated referring to a previously reported method [29]. Briefly, 100 mg of HD conjugates was dissolved in 5 mL formamide by gentle heating. Different amount of DOX·HCl were dissolved in 2 mL of DMF containing TEA (1.3 mole ratio of DOX·HCl). The two solutions were well mixed by vortexing, transferred into a pre-swollen membrane with a molecular weight cutoff of 8000–12,000 kDa (Spectrapor, Rancho Dominquez, CA, USA) and dialyzed against distilled water to remove the unloaded drug, DMF, TEA, formamide and triethylammonium chloride (TEA·HCl), and then lyophilized. The freeze-dried products were suspended in distilled water at 37 °C for 24 h, and the dispersed solution was sonicated three times using a probe-type sonifier (Sonics Ultrasonic Processor, VC750, Newtown, CT, USA) at 150 W for 2 min each to obtained DOX-loaded HD nanoparticles.

The contents of DOX incorporated in the HD nanoparticles were determined using a UV-VIS spectrophotometer (Lambda 20, PerkinElmer, Waltham, MA USA). Lyophilized DOX-loaded HD nanoparticles (3 mg) were dissolved in 3 mL of formamide to obtain clear solutions and DOX levels were quantified by UV absorption at 479 nm. The drug entrapment efficiency and drug loading content of DOX was calculated from Equations (1) and (2)

(1) $$LE=\;\frac{W\text{a}}{W\text{d}}\;\times 100\%$$

(2) $$LC=\;\frac{W\text{a}}{W\text{np}}\;\times 100\text{\%}$$

where *LE* is loading efficiency, *LC* is loading content; *W*_a_ is the amount of drug (mg) found in the drug-loaded nanoparticles, *W*_d_ is the amount of drug (mg) initial added into the system and *W*_np_ is the amount of drug-loaded nanoparticles.

### 3.5. Doxorubicin Release from HD Nanoparticles

*In vitro* drug release was performed in physiological saline solution (PBS, pH 7.2). Two milligrams of lyophilized DOX-loaded HD nanoparticles were dispersed in 2 mL of PBS and sonicated to obtained pellucid solutions. The dispersed drug-loaded nanoparticle solutions were transferred into cellulose ester membrane tubes (molecular weight cutoff = 8000 Da), which were then incubated in 100 mL vials containing 50 mL PBS. The vials were gently shaken at 37 °C in a water bath at 100 rpm/min. The system was protected from light. At predetermined sampling times, the whole medium was removed and replaced by fresh PBS so as to maintain a sink condition. The amount of DOX released from nanoparticles was determined with a UV-VIS spectrophotometer at 479 nm. The fractional release of the drug was calculated as a function of time (up to 20 h). All the data points are averages of three determinations.

### 3.6. Cell Culture

HeLa (human epithelioid cervical cancer cell line) was routinely cultured in RPMI-1640 medium (Life Technologies, Shanghai, China) and equilibrated with 5% CO_2_, and 95% humidity at 37 °C. The medium was supplemented with 10% heat-inactivated fetal bovine serum, 100 U/mL penicillin G and 100 mg/mL streptomycin.

### 3.7. Preparation of Fluorescein Isothiocyanate (FITC)-Labeled HD Conjugates

To label the HA-DOCA conjugates with FITC, HD conjugate (0.5 g) was suspended in 100 mL of bicarbonate buffer (0.1 M Na_2_CO_3_, 0.1 M NaHCO_3_, pH 9.5), 50 mg FITC in absolute ethanol at the concentration of 5 mg/mL was added, and the suspension was stirred for 24 h at room temperature. The reaction mixture was sonicated three times to obtain FITC-loaded HD nanoparticles. Then the nanoparticles suspension was dialyzed for three days against 20% ethanol with three separate medium exchanges to remove unlabeled FITC. The final FITC-labeled HD conjugates were obtained by freeze drying. The experiment was protected from light and the obtained labeled HD conjugates were stored in the dark.

### 3.8. In Vitro Cellular Uptake of Nanoparticles

HeLa cells were seeded into 96-well black plates (Costar, St. Louis, MO, USA) at 4 × 10^3^ cells/well and cultured in 200 μL RPMI-1640 at 37 °C in 5% CO_2_ until 80% confluence was achieved. The cultured medium was changed with fresh medium that contained FITC-labeled nanoparticles at a particle concentration of 0.01 to 0.2 mg/mL. At predefined periods of incubation at 37 °C, the suspension was removed and the wells were washed three times with 50 μL of PBS to eliminate traces of nanoparticles left in the wells. After that, 50 μL of 0.5% Triton X-100 in 0.2 N NaOH was added to the sample wells to lyse the cells. The fluorescence intensity of each sample well was measured in a fluorescence microplate reader (FL600, Bio-Tek, Vermont, NE, USA) using an excitation wavelength of 495 nm and an emission wavelength at 520 nm. Uptake efficiency was expressed by Equations:

(3) $$\text{Uptake Efficiency }\left(\%\right)=\;\frac{W\text{sample}}{W\text{total}}\times 100\%$$

where *W*_sample_ is the amount of FITC associated with HeLa cells, and *W*_total_ is the total amount of FITC present in the feed nanoparticle suspension.

### 3.9. The in Vitro Cytotoxicity Assay

The toxicity of blank HD nanoparticles, DOX-loaded HD nanoparticles and free DOX (control to the DOX-loaded nanoparticles) against HeLa cancer cells was investigated by the MTT assay. MTT is a yellow, water-soluble, tetrazolium salt. The mitochondrial enzymes in viable cells are able to convert it into a water-insoluble purple formazan by reductive cleavage of the tetrazolium ring [38]. Formazan crystals, then, can be dissolved in an organic solvent such as dimethylsulphoxide (DMSO). The amount of the formazan in the solution is quantified by measuring the absorbance at 490 nm, which correlates to the number of living cells [39].

The cells were harvested by trypsinization, and resuspended to a concentration of 3 × 10^4^ cells/mL in fresh culture medium. The cells were then seeded into 96-well flat bottomed tissue-culture plates at 3 × 10^3^ cells/well and incubated for 24 h in a humidified atmosphere of 5% CO_2_ at 37 °C. Then, the medium was replaced with the fresh medium containing DOX-loaded nanoparticles or free DOX at the final drug concentration from 1 to 10 µg/mL. After predefined time periods (24–72 h) of incubation at 37 °C, 20 µL of MTT solution (5 mg/mL in PBS, pH 7.4) was added into each well and plates were incubated at 37 °C for 4 h. The culture solution was removed and 200 µL DMSO was added in each well and vigorously shaken to dissolve any formazan crystals formed. The absorbance at 490 nm was measured in a microplate reader (BIO-RAD, model 550, Philadelphia, PA, USA) using DMSO blank. Three replicates were measured for each sample, the mean value of the three was used as the final result. Blank nanoparticles (non-loaded DOX) at the concentration of 0.2 mg/mL were also tested as vehicle controls. Cytotoxicity was expressed as percentage of cell viability calculated from the ratio between the number of cells treated with the different nanoparticle formulation (blank and drug-loaded nanoparticles) and that of non-treated cells (Control).

### 3.10. Statistical Methods

The assays were performed at least in triplicate on separate occasions. The data collected in this study are expressed as the mean value ± standard deviation. In all cases, *p* < 0.05 was considered significant, which is noted in figures with asterisks.

## 4. Conclusions

Our results demonstrate that the degree of substitution of DOCA of HA significantly affects the DOX loading efficiency and drug release behavior of drug-loaded HD nanoparticles. The drug loading efficiency can be enhanced by increased of feed weight ratio of DOX to HD and the release behavior of DOX exhibited a triphasic sustained pattern, in which the initial burst and release rate decreased with the increasing of DS. Furthermore, the cellular uptake of fluorescent HD nanoparticles is time- and concentration-dependent and correlates to the particles size which is affected by the DS. The results show that drug-loaded HD nanoparticles are more cytotoxic against cancer cells than free DOX at longer incubation periods. These data highlight the potential of HD nanoparticles for therapeutic applications as they improve the solubility and anti-cancer activity of DOX.
